# Supplementary material for: Co-expression analysis to identify key modules and hub genes associated with COVID-19 in platelets
Source: BMC Med Genomics. 2022 Apr 14;15:83. doi: 10.1186/s12920-022-01222-y (PMC9008611; doi:10.1186/s12920-022-01222-y)
Supplement: Supplementary file 1 — Additional file 1. Figs. S1–S3. [file 12920_2022_1222_MOESM1_ESM.pdf]

Coexpression Analysis to Identify Key Modules and Hub Genes Associated with COVID19 in Platelets

Supplementary Information

Ahmed B. Alarabi[1,+], Attayeb Mohsen[2,+], Kenji Mizuguchi[2,3], Fatima Z. Alshbool[1], and Fadi T. Khasawneh[4,*]

[1]Department of Pharmacy Practice, Irma Lerma Rangel College of Pharmacy. Texas A&M University, Kingsville, Texas, USA;

[2]Laboratory of Bioinformatics, Artificial Intelligence Center for Health andBiomedical Research (ArCHER), National Institutes of Biomedical Innovation,Health and Nutrition, 7-6-8 Saito-Asagi, 567-0085, Ibaraki, Osaka, Japan;

[3]Institute for Protein Research, Osaka University, 3-2 Yamadaoka, 567-0085, Suita, Osaka, Japan;

[4]Department of Pharmaceutical Sciences, Irma Lerma Rangel College of Pharmacy, Texas A&M University, Kingsville, Texas, USA.

[*]Correspondence to- Fadi T. Khasawneh, fkhasawneh@tamu.edu

[+]These two authors contributed equally to this work

Supplementary Figure 1

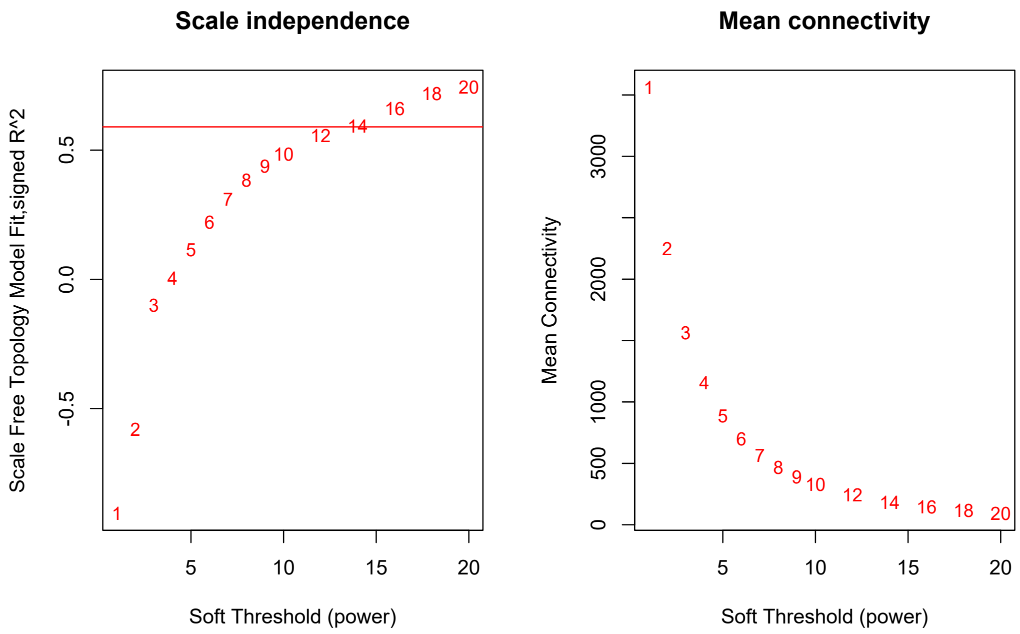

Supplementary Figure 1: Network Soft threshold

Supplementary Figure 2

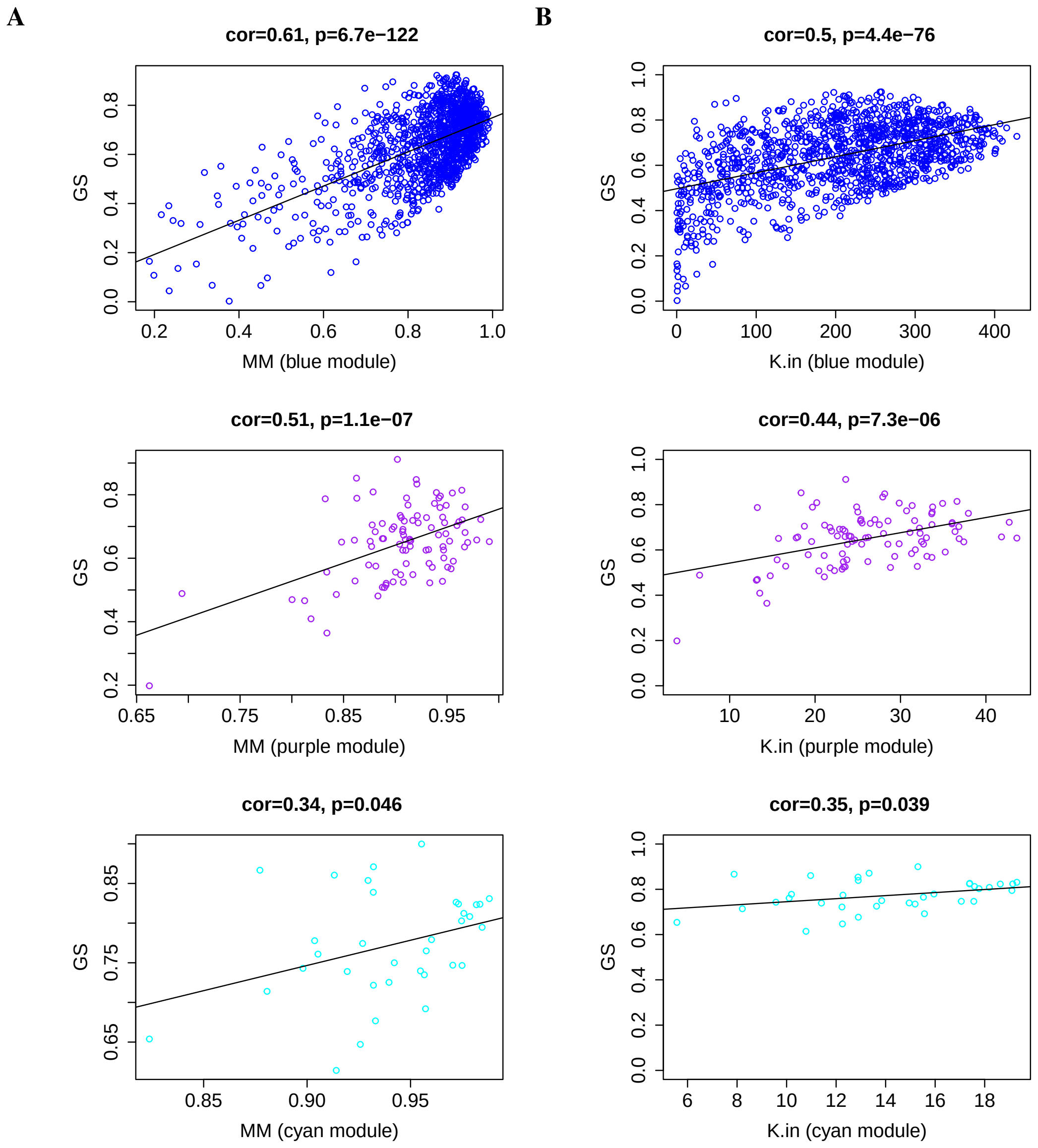

Supplementary Figure 2: Module Membership and gene significance in other modules.

Supplementary Figure 3

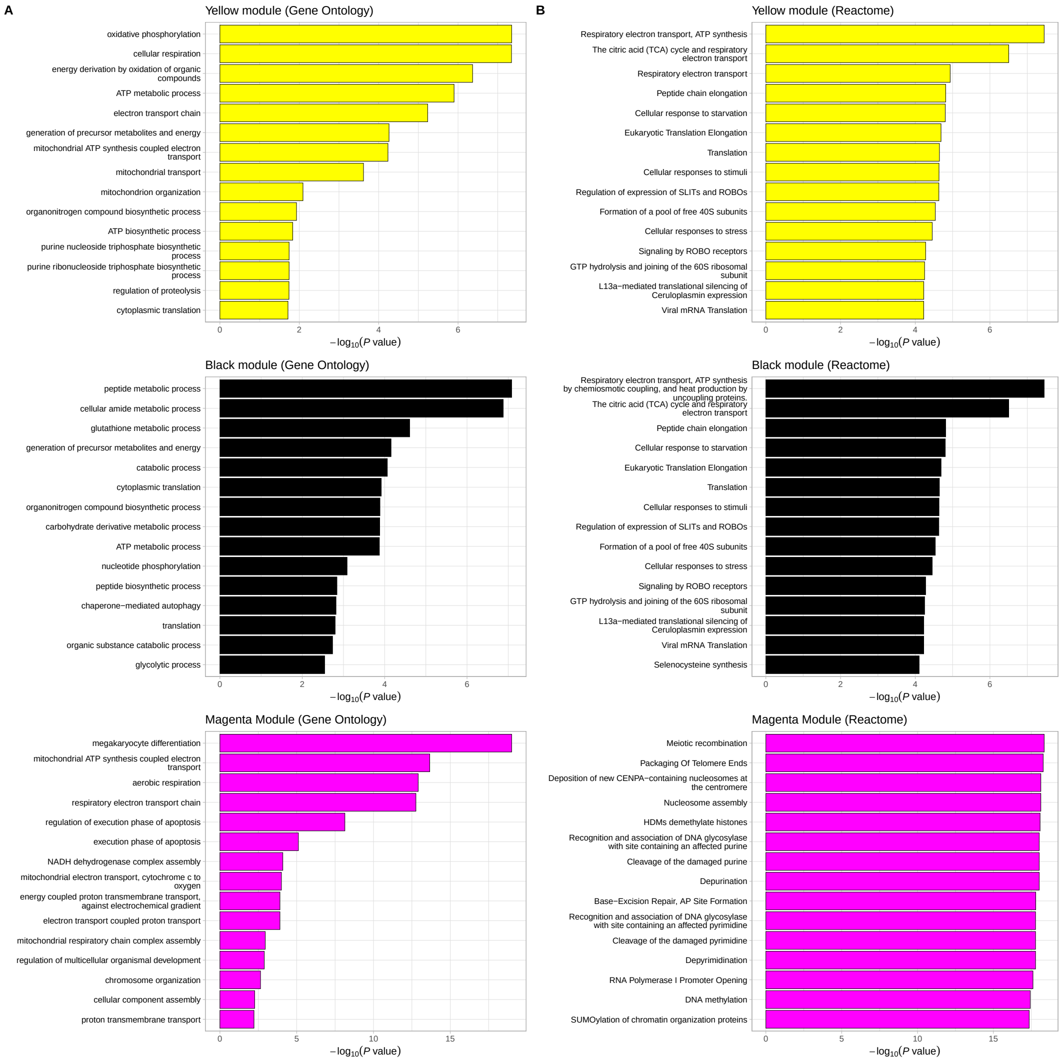

Supplementary Figure 3: Detailed enrichment analysis. A; Gene Ontology of biological function. B; Reactome pathways enrichment.

Supplementary Tables

Supplementary Table S1: Color Modules: Lists the genes and their corresponding modules (in colors) assigned by WGCNA.

Supplementary Table S2: Eignegenes: The Eignegenes of all WGCNA modules.

Supplementary Table S3: Gene significance: lists gene significance in COVID-19 computed in WGCNA (correlation of gene expression to COVID-19 phenotype).

Supplementary Table S4: Intra-modular connectivity: lists the genes network parameters; kTotal: total connectivity, kWithin: intramodular connectivity; KOut: extra-modular connectivity; kDiff: the difference of the intra- and extra-modular connectivities.

Supplementary Table S5: Hubgenes cross check: with Disgenet database.
